# A Town Enslaved by the Cocaine Habit

**Published:** 1897-03

**Authors:** 


### A TOWN ENSLAVED BY THE COCAINE HABIT.

It is reported in the daily papers that so large a number of the inhabitants of the town of Manchester, Conn., have become slaves to the cocaine habit, that in the opinion of an "experienced physician," "the future prosperity of the town is involved." The trouble arose from a preparation of cocaine and menthol, which a druggist in the town compounded about a year ago, and exploited as a remedy for asthma. So popular has this "snuff" become that people are seen on the streets and in dark corners at public entertainments, indulging their passion for the compound. According to the report, the druggists, although getting rich by the sale of the compound, are so bothered by "well-known men and women," who ring them up at all hours of the night to get a supply, threatening to break into the store if their wants are not attended to, that they have become prime movers in an effort to check the evil habit. Large numbers of the people are apparently running into debt to provide themselves with this expensive drug, and, if the story is true, we must acquiesce in the reported remark of a druggist, who says, "Their condition is pitiable." A drug habit which will drive "well-known men and women" to threaten violent entrance into drug-stores must indeed be a difficult problem for the town authorities to cope with.—*Boston Medical and Surgical Journal.*

